# Inferences about population dynamics from count data using multistate models: a comparison to capture–recapture approaches

**DOI:** 10.1002/ece3.942

**Published:** 2014-01-20

**Authors:** Elise F Zipkin, T Scott Sillett, Evan H Campbell Grant, Richard B Chandler, J Andrew Royle

**Affiliations:** 1 USGS Patuxent Wildlife Research Center12100 Beech Forest Rd., Laurel, Maryland, 20708; 2 Migratory Bird Center, Smithsonian Conservation Biology Institute, National Zoological ParkMRC 5503, Washington, District of Columbia, 20013; 3 USGS Patuxent Wildlife Research Center, Conte Anadromous Fish LaboratoryTurners Falls, Massachusetts, 01376; 4 Warnell School of Forestry and Natural Resources, University of GeorgiaAthens, Georgia

**Keywords:** Black-throated blue warbler (*Setophaga caerulescens*), N-mixture model, state-space model, structured populations

## Abstract

Wildlife populations consist of individuals that contribute disproportionately to growth and viability. Understanding a population's spatial and temporal dynamics requires estimates of abundance and demographic rates that account for this heterogeneity. Estimating these quantities can be difficult, requiring years of intensive data collection. Often, this is accomplished through the capture and recapture of individual animals, which is generally only feasible at a limited number of locations. In contrast, N-mixture models allow for the estimation of abundance, and spatial variation in abundance, from count data alone. We extend recently developed multistate, open population N-mixture models, which can additionally estimate demographic rates based on an organism's life history characteristics. In our extension, we develop an approach to account for the case where not all individuals can be assigned to a state during sampling. Using only state-specific count data, we show how our model can be used to estimate local population abundance, as well as density-dependent recruitment rates and state-specific survival. We apply our model to a population of black-throated blue warblers (*Setophaga caerulescens*) that have been surveyed for 25 years on their breeding grounds at the Hubbard Brook Experimental Forest in New Hampshire, USA. The intensive data collection efforts allow us to compare our estimates to estimates derived from capture–recapture data. Our model performed well in estimating population abundance and density-dependent rates of annual recruitment/immigration. Estimates of local carrying capacity and per capita recruitment of yearlings were consistent with those published in other studies. However, our model moderately underestimated annual survival probability of yearling and adult females and severely underestimates survival probabilities for both of these male stages. The most accurate and precise estimates will necessarily require some amount of intensive data collection efforts (such as capture–recapture). Integrated population models that combine data from both intensive and extensive sources are likely to be the most efficient approach for estimating demographic rates at large spatial and temporal scales.

## Introduction

A key objective of wildlife research is to determine how demographic parameters change over space and time. Understanding reproduction, survival, and dispersal of animals is necessary to determine the status and viability of populations, and to identify critical or sensitive portions of a population. These vital rate estimates can then be used to parameterize matrix models, or more complicated life cycle-based models, to assess whether populations are declining and determine the life stages that have the greatest influences on population growth rates and are thus most sensitive to change (Caswell 2001; Barker et al. 2009). In an applied context, this information can help researchers and wildlife managers to determine optimal approaches for management and conservation activities including the life stages and demographic rates to target, when action is warranted (Williams et al. 2002).

Estimating demographic parameters requires years of intensive data collection, most commonly through capture–recapture methods (Williams et al. 2002). Capture–recapture models have a long history in ecology, starting with the Lincoln–Petersen model (Pollock 1991; Nichols 1992), which provided the first method for estimating population abundance, and the Cormack–Jolly–Seber (Cormack 1964; Jolly 1965; Seber 1965) models, which introduced approaches for estimating apparent survival (Lebreton et al. 1992) and immigration. Since that time, a number of models have been developed to improve upon inferences from capture–recapture techniques by accounting for individual heterogeneity (e.g., Burnham and Overton 1978; Pledger et al. 2003; Royle 2008; Gimenez and Choquet 2010; Cubaynes et al. 2012). The multistate models were developed to estimate state-specific demographic rates with the assumption that life stage (and sex) frequently influences survival, reproductive, and dispersal probabilities (Arnason 1972;: Nichols et al. 1992; Schwarz et al. 1993; Nichols et al. 1994; Lebreton and Pradel 2002). The continued relevance of capture–recapture methodologies has led to a host of recent advances, including those focused on spatially explicit methods (Efford et al. 2009; Royle et al. 2013), state misclassification (Kendall et al. 2003), state uncertainty (Pradel 2005), disease dynamics (Conn and Cooch 2009), density dependence (Lebreton & Gimenez 2013), and the incorporation of telemetry data (Powell et al. 2000; Sollmann et al. 2013) as well as other auxiliary information (Pollock 2002).

While capture–recapture is the most direct way to attain detailed demographic information, these data can be expensive to collect and challenging to obtain at large spatial scales (Williams et al. 2002). Many capture–recapture surveys are therefore limited to a small number of locations, often providing information for just one species in a single region. As the pressure on wildlife populations increases, inexpensive and efficient approaches for estimating demographic rates that can be applied at large spatial and temporal scales are needed.

We build on N-mixture models (Royle 2004) to demonstrate an approach for estimating demographic rates using only state-structured count data while accounting for the imperfect detection of individuals during sampling. Our framework is based on the open population extension of the N-mixture model by Dail and Madsen (2011), which models abundance by separately accounting for apparent survival and population growth (reproduction and immigration) from count data over time. Zipkin et al. (2014) extended the Dail and Madsen (2011) model to obtain estimates of state-specific survivorship, immigration, and recruitment using only count data where individuals can be appropriately identified as belonging to one of a number of discrete states. Here, we expand this approach to incorporate data where the state is uncertain and apply our model to estimate sex-and (life) stage-specific survival as well as density-dependent recruitment rates for a population of black-throated blue warblers (*Setophaga caerulescens*). We use data collected on breeding grounds in the Hubbard Brook Experimental Forest in New Hampshire, USA. This warbler population has been studied extensively and, as a result, detailed information is available from capture–capture data (Sillett and Holmes 2002; Holmes 2007; Townsend et al. 2013), which we use for comparison.

## Materials and Methods

### Study system

Long-term demographic data on black-throated blue warblers were collected in the Hubbard Brook Experimental Forest, New Hampshire, U.S.A. (43°56′, 71°45′). This 3160 ha forest is part of the 3039 km^2^ White Mountain National Forest and is comprised of unmanaged, relatively mature, second-growth northern hardwood (Holmes 2011). Black-throated blue warblers, a sexually dichromatic passerine, were studied on three plots: from 1986 to 2010, a 65 ha, middle elevation site at 600 m above sea level, and from 1997 to 2010, a 85 ha low elevation site at 250–400 m, and a 35 ha high elevation site at 700–850 m above sea level. The three plots were separated from one another by at least 500 m. Habitat quality and warbler density increases with elevation at Hubbard Brook (Rodenhouse et al. 2003) such that the three plots were designed to contain roughly the same number of warbler pairs. Adult warblers were captured with mist nets and uniquely marked with colored leg rings. Birds were aged using plumage characteristics (Pyle 1997) as either yearlings in their first breeding season or older adults. Warbler territories were visited every two days to resight individuals and to monitor breeding activity. A small number of birds eluded capture throughout the breeding season in some years. In such cases, unmarked males were aged by visual observation of plumage characters (Graves 1997) when possible; unmarked females were not aged. Further details about field methods are provided in Sillett and Holmes (2002, 2005) and Townsend et al. (2013).

We collapsed this detailed capture–recapture dataset into stage-and sex-specific counts per year. We included the counts of individuals that were observed but not captured, such that we had the total annual count (e.g., number of unique individuals observed at least once from May to August in a given year) for six groups: yearling (age one) males, yearling females, adult (age two and older) males, adult females, males of unknown age, and females of unknown age. The number of males and females where age was unknown comprised a small portion of the data (<1% of males and 8% of females).

### Population model

**Figure 1:**
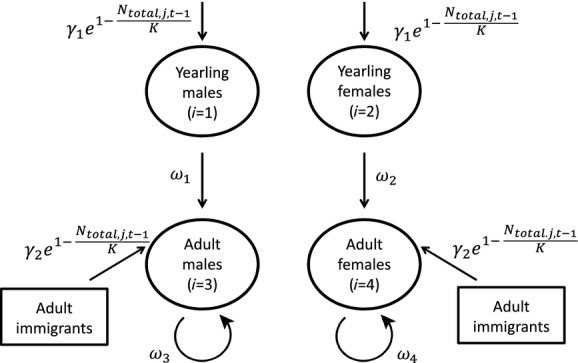
Diagram for the black-throated blue warbler (*Setophaga caerulescens*) population model. The parameters *ω*_1_–*ω*_4_ (state-specific annual survivorship), *γ*_1_ and *γ*_2_ (per capita rates of yearling and adult recruitment into the population), *N*_*j*,*t*−1_ (local population size in plot *j* at the previous time step), and *K* (carrying capacity) are all estimated using annual state-specific count data from the sampling plots.


Our model builds on Zipkin et al. (2014), which demonstrates how state-specific demographic rates and abundance can be estimated from multistate count data. For the black-throated blue warbler population, our interest lies in estimating the stage-(yearling or adult) and sex-specific survival rates and the annual number of yearlings and adults that recruit to the population. We do this by assuming that abundance, *N*_*i,j,t*_, of each state *i* at plot *j* in year *t* is dependent only on abundance of the local population in the previous year, *t*−1. Thus, we assume that dynamics in the three plots (high, middle, and low elevation) are independent of one another. We define four states of the warbler population (Fig. 1): yearling males (*i *=* *1), yearling females (*i *=* *2), adult males (*i *=* *3), and adult females (*i *=* *4). Because we have no information prior to the first year of sampling, we model abundance for each state in year *t *=* *1 as *N*_*i,j*,1_ ˜ Pois (*λ*_i_), where *λ*_i_ is the expected abundance of each state *i*, which we assume to be constant across the three plots. For all other years of sampling (*t* ≥ 2), we model abundance by considering (1) the annual number of individuals that survive (*S*) and return to the plot after migrating south for the winter and (2) the new individuals that are gained (*G*) to plots through reproduction or immigration, which is assumed to be a density-dependent process based on local abundance in the previous year. The number of surviving individuals, *S*_*i,j,t*_, in state *i* at time *t* at plot *j* is modeled as:

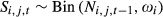


where *ω*_*i*_ is the survival probability for each of the four *i* states. To model the annual number gained at each plot, we considered the natural history of this population. Individuals that fledge from these plots rarely return to the exact same plot; thus, yearlings that create breeding territories in each of the *j* plots are technically immigrants, at least on this very local scale (Sillett and Holmes 2005). Fecundity (number of fledglings produced) and interannual variations in population abundance are density dependent, and yearlings are more likely to shift territory locations compared with adults (Rodenhouse et al. 2003). As such, we assumed that the number of individuals gained annually, *G*_*i,j,t*_ is a density-dependent recruitment process that is the same for both sexes but possibly different for yearlings and adults where


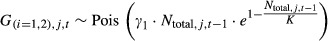



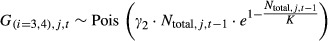


*N*_total,*j,t*−1_ is the total population size of all stages combined at plot *j* in year *t*−1, *γ*_1_ and *γ*_2_ are the per capita recruitment/immigration rates of yearlings and adults, and *K* (which is constant for all plots but could vary by year or plot if the quantity of data is sufficient) is the carrying capacity for each plot (Fig. 1). Thus, *γ*_1_·*N*_total_ and *γ*_2_·*N*_total_ are the expected number of yearlings and immigrating adults that enter the plot when the population is at carrying capacity, *K*. The annual state-specific abundances, *N*_*i,j,t*_, for *t* ≥ 2 are then:


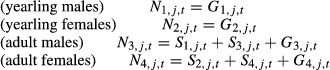


### Detection model: imperfect observation of state

We incorporated an additional component in this model to account for the fact that each of the *N*_*i,j,t*_ is detected imperfectly during the sampling process. As described above, our data, *n*_*i,j,t*_, arise from six states: the four states described in the model as well as males of unknown age (*i *=* *5) and females of unknown age (*i* = 6). We thus have two processes affecting the data recorded during sampling: the probability of detecting an individual and the probability of correctly classifying its state (conditional on detection). We specified the detection processes separately for males and females based on the results of Sillett and Holmes (2002), which suggest that detection may vary by sex. Let *p*_1_ be the probability of detecting a male and *c*_1_ be the probability of being able to correctly classify that individual as either a yearling or an adult. Then, the detection model for the male states can be specified for plot *j* in time *t* and repeated measure *k* as:


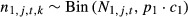



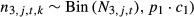



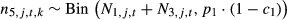


An analogous model is used for the female states where *p*_2_ is the detection probability of females and *c*_2_ is the probability of being able to classify the stage of that individual. This model implicitly assumes that errors in the detection process arise only from a failure to detect individuals (e.g., no false positives or misclassifation of sex). In our model, there is only one sampling event (*k *=* *1), but the same approach can be used when *k *>* *1.

### Model analysis

We used Bayesian inference and MCMC as implemented in R and JAGS (Plummer 2003) to simulate posterior distributions of parameters. For the process model, we set vague priors for survivorship, *ω*_*i*_ ˜ Uniform (0,1), per capita recruitment/immigration, *γ*_1,2_ ˜ Uniform (0,30), initial state-specific abundance, *λ*_*i*_ ˜ Uniform (0,30), and carrying capacity, *K* ˜ Uniform (0,150). The upper bounds for *γ*, *λ*, and *K* parameters were well above biologically reasonable values. For the detection model, we only had data from a single annual survey event, which represented the total number of observed individuals within states for each breeding season. Although estimating a detection probability for an open population is possible with only one survey replicate over 25 years of data (Dail and Madsen 2011), the simulation results in Zipkin et al. (2014) suggest that the limited number of plots in our study could lead to imprecise parameter estimates. Therefore, we used informative priors on the detection probabilities of males, *p*_1_ ˜ Normal (0.926, 0.034)T(0,1), and females *p*_2_ ˜ Normal (0.869, 0.061)T(0,1), taken from annual detection/capture probabilities (mean and standard deviation) in Sillett and Holmes (2002) derived using a Cormack–Jolly–Seber model (CJS) on a subset of these data on the mid-elevation plot from 1986 to 1999. T(0,1) indicates that the distribution is truncated so that detection cannot be below zero or above one. We set vague priors on both the male and female classification parameters where *c* ˜ Uniform (0,1). We generated three Markov chains, each consisting of 20,000 iterations after a burn-in of 20,000 iterations, and we saved every 10th sample, such that we obtained 6000 posterior samples for each parameter. We checked convergence by examining plots of the posterior distributions and the variance within and between chains to ensure that the Brooks–Gelman–Rubin diagnostic (i.e., 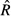 statistic) was <1.05 for all parameters.

## Results

**Figure 2:**
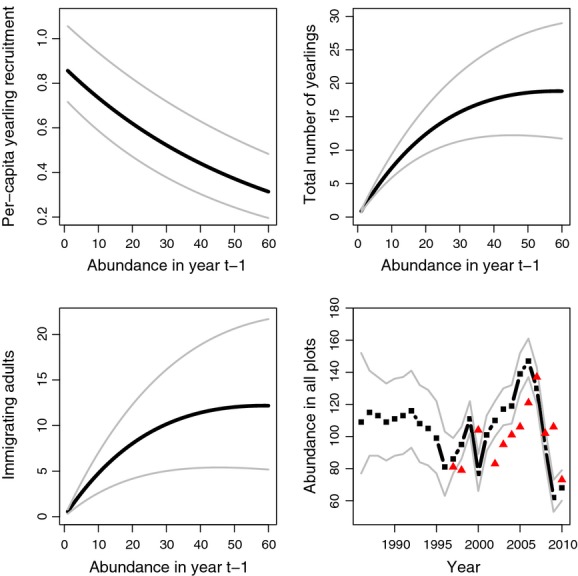
Per capita recruitment of yearlings (top left panel), the total number of yearlings that recruit (top right panel), and the total number of adults that immigrate (bottom left) into a plot in year *t* versus the plot population size in year *t−*1. The final panel (bottom right) shows the predicted population size of all three plots combined. The black lines represent the median estimates, and the gray lines show the 95% posterior range. The red triangles represent the total number of yearlings and adults in the three plots as estimated from territory maps for years when data on all plots were available.

**Figure 3:**
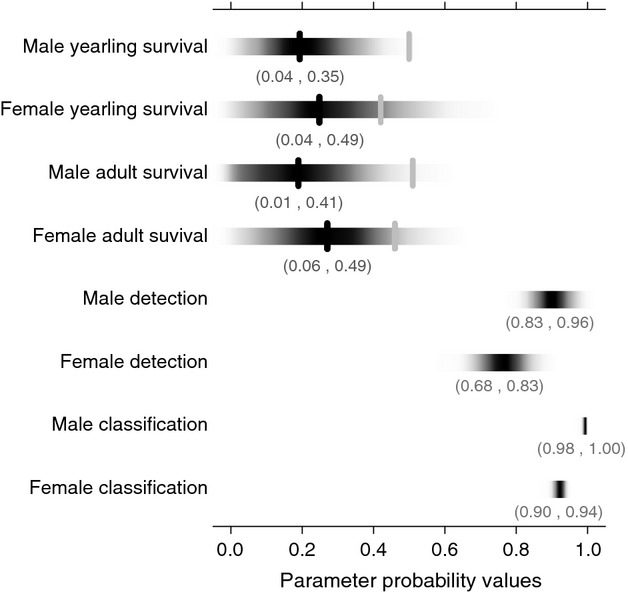
Parameter estimates for black-throated blue warbler survival probabilities for male and female yearlings (*ω*_1_ and *ω*_2_) and adults (*ω*_3_ and *ω*_4_), as well as sex-specific detection (*p*_1_ and *p*_2_) and classification (*c*_1_ and *c*_2_) probabilities (e.g., probability of being able to identify males and females as either a yearling or an adult) where darker values indicate greater posterior mass. The 95% confidence intervals are also shown for each parameter. The median values for the survival probabilities (where the intervals are larger) are indicated with a black line. The gray lines indicate the average state-specific survival estimates produced with a Jolly–Seber model using the individual encounter histories.

Some parameter estimates produced by the state-structured model, such as those related to fecundity and carrying capacity, were consistent with capture–recapture studies as well as to inferences from other black-throated blue warblers studies in the region (Fig. 2), whereas other parameter estimates, such as survival, were less consistent (Fig. 3). The carrying capacity, *K*, of the plots had a median value of 58.7 warblers (95% confidence interval, CI: 45.4–75.4). This is consistent with estimates in Rodenhouse et al. (2003; Fig. 1) showing that population growth rate is near zero when density of adults was around 10–11 breeding individuals per 10 hectare, which translates to 62.5–71.5 individuals on our middle elevation – and medium habitat quality – plot. Although directly comparing the per capita rate of yearling recruitment from our model (top panels of Fig. 2) with available estimates of fecundity (e.g., young fledged per breeding pair) is difficult, these results are consistent in the negative impact of high density (Sillett and Holmes 2005). Our estimates of *γ*_1_ (median: 0.16; 95% CI: 0.13–0.20) suggest that a breeding pair produces about 0.64 yearlings when the population is at carrying capacity (e.g., per capita recruitment of both male and female yearlings at *K* is 2*γ*_1_ and thus per capita recruitment per breeding pair is 4*γ*_1_). Rodenhouse et al. (2003; Fig. 2) estimated that approximately 2 fledglings were produced per breeding pair at similar densities (10 breeding individuals per 10 hectares). This would indicate that approximately one-third of individuals survive from fledging to their first breeding season, similar to the 0.3 value that Sillett and Holmes (2005) used to approximate observed warbler dynamics. At carrying capacity, the expected number of yearlings (male and female) recruiting to a plot is approximately 18.8 (2*γ*_1_ · *K*), again similar to Sillett et al. (2000) who showed that the number of yearling males entering a plot ranged from 2 to 14 based on the previous year's fecundity (i.e., assuming an equal sex ratio, we would expect about 4–28 total yearlings annually). Adult immigration at carrying capacity was approximately 12.2 individuals (2*γ*_2_ · *K*; *γ*_2_ median: 0.10, 95% CI: 0.06–0.15). The recruitment rates of yearlings and adults suggest that more yearlings move into the plots than immigrating adults, which is consistent with existing knowledge of the system (Rodenhouse et al. 2003). The temporal patterns of abundance as estimated in our model were consistent with those produced using territory maps and tended to capture observed variations well (Fig. 2).

Estimates of sex-and stage-specific survival from our model (Fig. 3) were lower and had larger confidence intervals than those estimated using the complete capture–recapture information. The 95% confidence intervals for yearling females (*ω*_2_ median: 0.25, 95% CI: 0.04–0.49) and adult females (*ω*_4_ median: 0.27, 95% CI: 0.06–0.49) contained the values produced using a Jolly–Seber model of the capture–recapture data over the same time period (0.42 [SE: 0.04] for yearling and 0.46 [SE: 0.02] for adult females). However, survival estimates for males were more severely underestimated, with the 95% CI of yearling (*ω*_1_ median: 0.19, 95% CI: 0.04–0.35) and adult (*ω*_3_ median: 0.19, 95% CI: 0.01–0.41) survivorships not containing the capture–recapture estimates (0.50 [SE: 0.03] for yearling and 0.51 [SE: 0.02] for adult males). The low rates of survival combined with the overestimation of adult immigration suggest that our model may have had difficulty distinguishing between these two processes for this dataset.

The posterior distribution for the probability of male detection (*p*_1_ median: 0.89; 95% CI: 0.83–0.96) was slightly lower, but still similar to its prior; the posterior distribution for the detection probability of females (*p*_2_ median: 0.75; 95% CI: 0.68–0.83) was more diffuse and lower than the prior (Fig. 3). This result may not be surprising because the data include individuals of unknown age (*i *=* *5,6) that could not be included for capture–recapture analysis, and more of those individuals are female than male (8% compared with less than 1%). Estimates for classification probabilities were quite high for both males (95% CI: 0.98–1.0) and females (95% CI: 0.90–0.94), with males having a slightly higher probability of correct classification (Fig. 3). This is consistent with our expectation, given that noncaptured females cannot be accurately aged by visual observations (T. S. Sillett, pers. obs.).

## Discussion

The state-structured models developed in Zipkin et al. (2014) and presented in this article represent an extension of the Dail and Madsen (2011) open population model as well as an extension of multistate models to populations for which data on individual encounter histories are not available (Table 1). Link et al. (2003) developed a similar age-structured approach for estimating demographic rates from aggregate count data. As with our approach, their method assumed a first-order Markovian structure to estimate annual age-specific survival probabilities in a population of whooping cranes. In their case, whooping cranes were censused implying that there were no omissions during the sampling process and that detection probabilities did not need to be incorporated. To our knowledge, all other multistate models in the literature were developed for use in marked populations. Our state-structured model, which requires only count data, provides an inexpensive method for obtaining inferences on state-specific demographic rates, while accounting for imperfect detection. The relative ease of collecting count data suggests that our modeling framework could be applied on broader spatial scales than is possible with intensive capture–recapture techniques. However, the approach can have drawbacks in that estimates of demographic rates and population abundance could be inaccurate and/or imprecise if data are limited. For example, Zipkin et al. (2014) conducted a number of simulations for a theoretical two-stage population and found that estimates of recruitment were biased low while estimates of juvenile survivorship were biased high when only two years of data were available even with a large numbers of sampling locations (e.g., 100 sites). Yet, their simulation results also demonstrated that median parameter estimates were unbiased when 20 years of data were available at only five locations. In that scenario, precision on all parameters was improved with three sampling replicates as compared to only one. These results indicate that parameter estimates can be improved by increasing the length of the time series and the number of replicate surveys, and not necessarily by the addition of more sampling locations. This suggests that our 25 years of black-throated blue warbler data should be adequate for inference even though the number of sampling plots was limited.

**Table 1:** Data structures and possible population models used to estimate abundance and demographic rates (in some cases) for instances where samples consist of live individuals.

Data structure	Population (stage/class) structure	
	One stage/class	Multiple stages/classes
Marked individuals	Jolly–Seber models and Cormack–Jolly–Seber models	Multistate models
	(Cormack 1964; Jolly 1965; Seber 1965)	(Nichols et al. 1992; Schwarz et al. 1993; Lebreton and Pradel 2002)
Unmarked individuals	N-mixture models	State-structured open population N-mixture models
	(Royle 2004; Dail and Madsen 2011)	(Zipkin et al. 2014; model presented in this article)

To improve our results and because the information was available, we included strong priors on detection probabilities. As expected, parameter estimates were much more imprecise when the model was rerun with an uninformative prior on detection (results not shown) and model runs did not converge well, indicating that the interpretation of our results is sensitive to our decision to use an informative prior. Our model of the black-throated blue warbler population produced less accurate and precise estimates of demographic rates as compared to a similar state-structured model in Zipkin et al. (2014) of a northern dusky salamander population. This is likely due to the relatively simple life cycle of these salamanders in which population abundances are largely driven by local dynamics and individuals are fairly sedentary. If movement rates are high, as in our population of warblers (Cline et al. 2013), then estimates of apparent survival can be confounded with local immigration because individuals are not identifiable. Similarly, black-throated blue warblers have low natal philopatry. Nearly all yearlings that recruited into our study plots were likely fledged outside of the plots such that the population dynamics among sites are not completely independent. Nevertheless, annual fecundity of warblers breeding on our study plots is positively correlated with the number of yearling recruits in the following spring (Sillett et al. 2000), indicating that demographic variation documented on our Hubbard Brook study plots is representative of breeding season dynamics occurring in the larger White Mountains region of New Hampshire and possibly areas beyond that (Jones et al. 2003). Our model produced similar patterns of abundance as compared to estimates from territory maps (Fig. 2). This suggests that state-structured models derived from count data can be useful for characterizing the general temporal patterns to estimate trends even when it is not possible to estimate all demographic parameters with complete accuracy.

During development of our black-throated blue warbler model, we considered many forms of density dependence on the number of individuals gained (*G*) to the population. We settled on the model presented here because it represented a biologically plausible description of black-throated blue warblers, demographic parameter estimates were insensitive to choice of priors (on those parameters), and it had the lowest deviance information criterion (DIC = 2119). However, we note that another model which assumed that adult immigration was not density dependent was the second choice (DIC = 2218). In this model, annual survival probabilities were slightly higher (median values: *ω*_1_ = 0.20, *ω*_2_ = 0.29, *ω*_3_ = 0.36, *ω*_4_ = 0.38), carrying capacity was lower (median *K *=* *51.7), and the number of immigrating adults, which was constant, was lower at carrying capacity (expected number of new adults annually: 7.4). The expected number of yearlings to recruit into the population at carry capacity, 18.1 individuals, was quite similar. A comparison of these models indicates that the choice of model structure can play an important role in the interpretation of results and that it can be difficult to distinguish between mechanistic processes when only count data are available. Indeed, parameter estimates produced by open N-mixture models are model-based by definition (Dail and Madsen 2011; Zipkin et al. 2014), and thus, the utility of results depends on how closely the data conform to the parametric assumptions of the model. For example, our model assumes that the number of individuals gained to the population is only dependent upon local abundance in the previous year, but it is quite possible that recruitment is dependent among a number of other factors such as abundance in adjacent locations and/or conditions during winter migration. Parameter estimates could be imprecise or even biased if data do not conform to the assumptions of the model. As such, it is important to consider the major processes governing dynamics within populations and to outline the assumptions made when estimating quantities from state-structured count data. Similarly, parameter identifiability should be explored during model development. In some cases, it may not be possible to estimate all parameters of interest.

In this article, we expanded the state-structured model of Zipkin et al. (2014) by incorporating data where the state cannot be observed perfectly. We demonstrated this approach in our detection model for the case where sex was known but life stage could not always be determined; but this method could be used when some individual states are unknown. It might also be possible to improve inferences on detection if observation models other than the binomial (i.e., the simple counting model) are available. For example, if data can be collected using a removal approach or distance sampling, a multinomial observation model could be used, which would likely result in better precision on estimates of detection and thus population abundance and demographic rates.

The use of capture–recapture techniques will continue to be important in estimating population abundance and demographic rates because of the high quality information that such methods can produce. Yet, capture–recapture cannot be the only approach for obtaining demographic parameter estimates due to its limited spatial extent and relatively high cost. While methodological advancements for unmarked populations – such as the one presented in this article – offer exciting alternatives, unmarked populations cannot provide the same detailed information as marked populations. Thus, the most comprehensive approaches to estimate population parameters on large spatial and temporal scales may be to integrate intensive data (e.g., capture–recapture) with extensive data (e.g., counts of unmarked individuals). Besbeas et al. (2002) and Brooks et al. (2004) developed integrated population models that use a combination of band recovery data and population indices to formulate more robust estimates of abundance and demographic rates than is possible using only the mark-recovery data. More recently, a number of studies have used integrated population models to explore local population dynamics (Barker et al. 2009; Schaub et al. 2009, 2012), determine the conservation status of threatened species (Schaub et al. 2009, 1964), and improve parameter estimates when data are limited (Schaub et al. 2007; Abadi et al. 2010). In most cases, capture–recapture or ring-recovery data provide the direct information for estimating demographic parameters. The results of our study suggest that it may be possible to use count data in broader ways within integrated models, especially if it is possible to obtain counts of individuals in relevant life states (e.g., sex and stages). This could lead to exciting opportunities for developing mechanistic-based integrated models for improved estimates of population processes and abundances.
